# Effects of quercetin on insulin-like growth factors (IGFs) and their binding protein-3 (IGFBP-3) secretion and induction of apoptosis in human prostate cancer cells

**DOI:** 10.1186/1477-3163-5-10

**Published:** 2006-04-06

**Authors:** Marati R Vijayababu, A Arunkumar, P Kanagaraj, J Arunakaran

**Affiliations:** 1 Department of Endocrinology, Dr. ALM Postgraduate Institute of BasicMedical Sciences, University of Madras, Taramani campus, Chennai-600 113, INDIA

## Abstract

**Background:**

Quercetin, the predominant flavonoid, has been reported to lower the risk of several cancers. This flavonoid found in onion, grapes, green vegetables, etc. has been shown to possess potent antiproliferative effects against various malignant cells. This study was designed to investigate its effects on insulin-like growth factors (IGFs) and their binding protein-3 (IGFBP-3) proteins secretion and also apoptosis induction in the human prostate cancer cell line, PC-3.

**Methods:**

We evaluated the secretion of IGF-I, -II and IGFBP-3 in quercetin treated cells by immunoradiometric (IRMA) method. Apoptosis was studied in quercetin treated cells by TUNEL and DNA fragmentation. Protein expressions of Bcl-2, Bcl-x_L_, Bax and caspase-3 were studied by western blot.

**Results:**

At a dose of 100 μM concentration, we observed increased IGFBP-3 accumulation in PC-3 cells conditioned medium with a dose dependent increase with 2 fold over a base line, and significantly reduced the both IGF-I and IGF-II levels. Apoptosis induction was also confirmed by TUNEL assay. Bcl-2 and Bcl-x_L _protein expressions were significantly decreased and Bax and caspase-3 were increased.

**Conclusion:**

These results suggest that the decreased level of IGFs could be due to the increased levels of IGFBP-3, because of the high binding affinity towards IGFs, thereby decreasing the cell proliferation. The increased level of IGFBP-3 was associated with increased pro-apoptotic proteins and apoptosis in response to quercetin, suggesting it may be a p53-independent effector of apoptosis in prostate cancer cells.

## Background

Much attention has focused on the growth hormones antagonists from the natural sources for the treatment of prostate cancer are being developed to block the activity of IGFs or to promote the activity of IGFBP-3; these agents may offer additional ways of stimulating apoptosis in malignant cancer cells. Quercetin, a flavonoid found in onion, grapes, green vegetables, etc. has been shown to possess potent antiproliferative effects against various malignant cells [1-3]. Recent report indicated that quercetin inhibited the proliferation of rat prostate cancer cells by interacting with IGF-I signaling pathway [4]. IGFBP-3 is a member of a family of specific high affinity binding proteins that modify the mitogenic actions of IGFs by regulating their access to the IGF-I receptor (IGFRI). There is still some ambiguity in determining the role of IGFBP-3 in regulating IGF actions, with studies showing both enhancement and inhibition of IGFs mitogenic effects [5,6]. However, IGFBP-3 has been shown to have IGFRI-independent anti-proliferative and proapoptotic effects. IGFBP-3 is expressed in many tissues including prostate [7] and others have demonstrated its growth inhibitory effects in human prostate cancer cells [8,9] as well as in IGFRI-null fibroblasts [10]. More recently, it has been suggested that the anti-proliferative effects of IGFBP-3 may be mediated via an induction of apoptosis. Indirect evidence has come from reports that an increase in IGFBP-3 expression is associated with the induction of apoptosis [11-15]. High levels of recombinant nonglycosylated IGFBP-3 can induce apoptosis in MCF-7 breast carcinoma cells, although under some conditions these levels of exogenous IGFBP-3 may induce apoptosis indirectly by sequestering anti-apoptotic IGFs from the IGFRI [14]. However, no induction of apoptosis was observed in Hs578T breast carcinoma cells exposed to exogenous IGFBP-3 alone [16], although in this study IGFBP-3 augmented the cellular response to apoptotic stimuli. Studies by Rajah *et al *[12] in prostate carcinoma cells and IGFRI-negative mouse fibroblasts showed an induction of apoptosis by both exogenous and endogenous IGFBP-3 and provide the strongest evidence of an IGFRI-independent pro-apoptotic role for IGFBP-3 in cancer cell growth. IGFBP-3 production was induced by potent growth inhibitory and pro-apoptotic agents including transforming growth factor β1 [TGF-β1; 14], retinoic acid [17], and tumor necrosis factor-a [18] with evidence that these agents mediate their cellular effects through IGFBP-3. Furthermore, IGFBP-3 is an established target of the tumor suppressor p53 [19,20].

The Bcl-2 family has been identified as an important regulator of mammalian cell death with both anti-apoptotic (*e.g. *Bcl-2 and Bcl-x_L_) and pro-apoptotic (*e.g. *Bax and Bad) members [21]. Members of this family regulate susceptibility to apoptosis, whereby over expression of Bcl-2 or Bcl-x_L _in relation to Bax promotes survival, but over expression of Bax accelerates cell death. Bax over expression in prostate cancer cells leads to induction of apoptosis [22]. These Bcl-2 related proteins play a role in the cell proliferation and apoptosis of prostate cancer [23], suggesting changes in the ratio of Bcl-2-related proteins may be an important determinant in the response of prostate cancer cells to apoptotic stimuli. Our previous study showed that quercetin reduce, IGF-I protein secretion in conditioned media, while IGFBP-3 was induced in prostate cancer cells [24]. In this study, the effect of quercetin on the induction of apoptosis in human prostate cancer cells, which is a p53 negative cell line, was examined.

As quercetin is more readily consumed worldwide, it is worth investigating the effects of quercetin on prostate cancer. While, IGFs are capable of enhancing, proliferation and inhibiting apoptosis in normal and malignant prostate epithelial cells by autocrine/paracrine actions, IGFBP-3 is found to be pro apoptotic. So the present study was aimed to investigate the effect of quercetin on IGFs and IGFBP-3 secretion in PC-3 cells.

## Materials and methods

Minimum Essential Medium (MEM), Fetal Bovine Serum (FBS), Trypan Blue and quercetin were purchased from Sigma Chemical Co., USA. Thymidine [^3^H] was purchased from BRIT, Mumbai. Other chemicals were obtained from Sisco Research Laboratories (SRL), India. All the chemicals used were extra pure and were of culture grade. The androgen-independent prostatic carcinoma PC-3 cell line was procured from National Center for Cell Science (NCCS), Pune, India. Quercetin was dissolved in DMSO. DMSO in culture media never exceeded 0.1% (v/v), the concentration known not to affect the cell proliferation and IGFBP-3 production. Cell viability was tested by Trypan Blue exclusion method. PC-3 cells were plated at 1 × 10^5 ^cells per well in 12-well plates in MEM containing 5% FBS. The growth inhibitory effect of quercetin was studied using Thymidine [^3^H] incorporation. For IGF-I, -II and IGFBP-3 assays, 1 × 10^6 ^cells were treated with quercetin (25, 50, 75 and 100 μM) in conditioned medium containing 0.1% Bovine serum albumin for 24 h and 48 h. The doses were selected based on of our previous studies [24].

### Cell proliferation

Cell proliferation was assessed by Thymidine incorporation method [25]. During the final 4^th ^h of quercetin treatment, 1 μCi/well [^3^H] thymidine-containing medium was added and incubated. Monolayers were rinsed twice with ice-cold saline and fixed with 1 ml/well ice-cold methanol-acetic acid mixture at 4°C for a minimum of 2 h. Cells were solubilized in 0.5 ml of SDS and 250 μl of each lysate was mixed with the scintillation fluid, before counting.

### Analysis of conditioned media for IGF-I, -II and IGFBP-3 secretion

IGF-I, -II and IGFBP-3 secretion in conditioned media of control and quercetin treated PC-3 cells were quantitated by immunoradiometrically (IRMA). Cells were seeded at 1 × 10^6 ^cells in each petri dishes and treated with quercetin for 24 h and 48 h. Conditioned media were collected 24 h and 48 h later and analyzed for IGF-I, -II and IGFBP-3. The results were expressed as ng/ml.

### Western blotting of Bcl-2, Bcl-x_L_, Bax and caspase-3

Cells were plated on 75 cm^2 ^flasks at density of 2 × 10^6 ^cells per flask, allowed to attach overnight and then exposed to quercetin for 24 h. Control cells were exposed to DMSO for 24 h. After the cells were washed three times with 33ice-cold PBS, then total proteins were extracted by adding 200 μl of cold lysis buffer (50 mM Tris-HCl, pH 7.4; 1 mM sodium Fluoride (NaF); 150 mM NaCl; 1 mM EDTA; 1 mM phenylmethylsulphonyl fluoride (PMSF) and 10 mg/ml leupeptin) to the cell pellets. After 30 min on ice the cell debris was pelleted by centrifugation at 10 000 × g for 30 min at 4°C. The proteins were quantified by Lowry's method [26]. The samples (50μg of total protein) were mixed with 5X sample buffer, containing 0.3 M Tris-HCl (pH 6.8), 25% 2-mercaptoethanol, 12% sodiumdodecyl sulphate (SDS), 25 mM EDTA, 20% glycerol, and 0.1% bromophenol blue. The mixtures were boiled at 95°C for 5 min, and subjected to 10–15% SDS-PAGE (polyacrylamide gel electrophoresis) at a constant current of 20 mA. Following electrophoresis, proteins on the gel were electro-transferred onto a PVDF membrane (Sigma, U.S.A.) in transfer buffer composed of 25 mM Tris-HCl (pH 8.9), 192 mM glycine and 20% methanol. The membranes were blocked with 20 mM Tris-HCl (pH 7.4), 125 mM NaCl, 0.2% Tween 20, 3% bovine serum albumin (BSA), and 0.1% sodium azide. The membranes were then immunoblotted with mouse monoclonal anti-human Bcl-2, Bcl-x_L_, Bax, caspase-3 and β-actin antibodies (generous gift from Dr. Ion V. Deaciuc, University of Louisville, Kentucky, USA). The membranes were washed and were incubated with horseradish peroxidase-labelled antimouse Rabbit IgG antibody at a dilution of 1:1000. The bands were developed using ECL kit (Perkin Elmer, USA) and density of the bands was detected with scanning densitometry.

### Flowcytometry analysis

Cells were plated at 1 × 10^6 ^cells in a 25 cm^2 ^flasks for 24 h. Cells were then washed with fresh media and treated with 50 and 100 μM concentration of quercetin. 24 h post-treatment, cells were trypsinized, and aliquots of 1 × 10^6 ^cells were suspended in 1 ml of fluorochrome solution (50 mg/ml propidium iodide, 1 mg/ml RNase A, 1.5% Triton X–100) for at least 1 h in the dark at 4°C. Cell cycle analysis was performed using a Beckman Vantage flow cytometer.

### DNA fragmentation

After 24 h and 48 h of treatment, the DNA was extracted from the cell lysate as follows. Both attached and floating cells were collected, washed with PBS and centrifuged at 1500 × g for 5 min to collect the cell pellet, which was resuspended in 0.5 ml of lysis buffer, transferred to a microfuge tube and incubated for 1 h at 37°C. To this, 4 μl of proteinase K (1 mg/ml) was added and the tubes were incubated at 50°C for 3 h. To each tube, 0.5 ml of phenol-chloroform-isoamyl alcohol (25: 24: 1) was added, mixed and centrifuged at 10,000 × g for 1 min to separate the DNA containing upper aqueous phase. To the resulting aqueous phase, 2 vol of ice-cold absolute ethanol and 1/10 vol of 3 M sodium acetate were added and incubated for 30 min on ice to precipitate DNA. DNA was pelleted by centrifuging at 10, 000 × g for 10 minutes at 4°C, the supernatant was aspirated and the pellet was washed with 1 ml of 70% ethanol. Then the DNA was quantified by UV-visible spectroscopy and electrophoresed in a 1.2% agarose gel containing ethidium bromide.

### TUNEL

DNA strand breaks in apoptotic cells were measured by terminal deoxynucleotidyl transferase mediated biotinylated UTP nick end-labeling (TUNEL) using an *in situ *cell death detection kit from Roche Molecular Biochemicals, Germany. Treated cells were harvested and fixed with 4% paraformaldehyde solution and incubated in a 0.1% Triton permeabilization solution on ice according to the manufacturer's instructions. Cells incubated with the solution without the terminal transferase was used as a negative control.

### Statistical analysis

The data were analyzed using the SPSS 7.5 Windows Students version software. For all the measurements, one-way ANOVA followed by Student's Newman Keuls (SNK) test was used to assess the statistical significance of difference between control and quercetin-treated. A statistically significant difference was considered to be at *P < 0.05*.

## Results

### Cell proliferation

**Figure 1:**
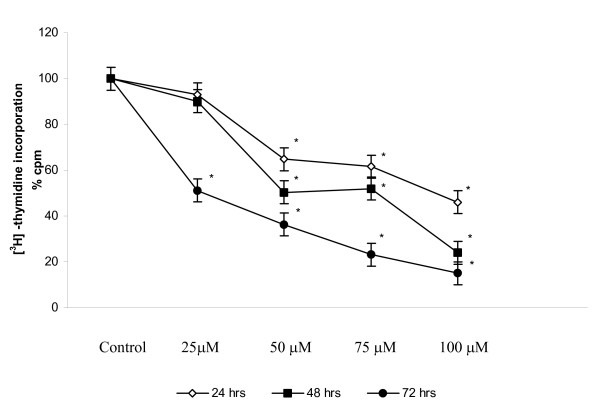
Effect of quercetin on PC-3 cell proliferation. 3 × 10^3 ^cells were plated in 24-well plates. After reaching 70–80% confluence, in the presence of 5% FBS, cells were treated with vehicle or with various concentrations of quercetin (25, 50, 75, and 100 μM) for 24 h, 48 h and 72 h. Proliferation of cells was quantitated by [^3^H] thymidine incorporation. Values represents % of control. Experiments were performed in triplicate, and SD was less than 10%. denotes the statistical significance at *P < 0.05 *level between control and quercetin treated using Student's Newman Keuls (SNK).

PC-3 cells showed a significant decrease in thymidine [^3^H] uptake. Fig.1 shows the kinetics of proliferation upto 0 – 72 h quercetin treatments, during which the thymidine uptake was decreased between 3- and 4- fold in quercetin treated cells. Time response data demonstrate 50% growth inhibition at 100 μM for 24 h.

### IGF-I, -II and IGFBP-3 level

After treatment of quercetin, the secretions of IGF-I,-II and IGFBP-3 levels were measured in conditioned media by immunoradiometric method. The secretion of IGF-I was significantly reduced in conditioned media of quercetin treated PC-3 cells. The IGF-II level was also significantly reduced in conditioned media of quercetin treated PC-3 cells. Interestingly, the secretion of IGFBP-3 by quercetin treated PC-3 cells was significantly increased in quercetin treated conditioned medium of both 24 h and 48 h treated experiments (Table-1).

### Protein expression of Bcl-2, Bcl-x_L_, Bax and caspase-3

**Figure 2:**
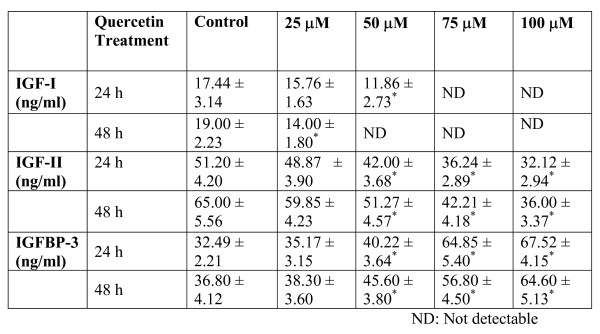
Effects of quercetin on Bcl-2, Bcl-x_L_, Bax and caspase-3 proteins expression in prostate cancer cell line (PC-3). PC-3 cells were treated with indicated concentrations of quercetin for 24 h. Cell lysates were analyzed by Western blotting. Blots were incubated with anti-Bcl-2, anti- Bcl-x_L_, Bax, caspase-3 and anti-β actin antibodies.

To investigate a possible intracellular mechanism for the observed increase in apoptosis, we examined the protein expression of the apoptotic intermediates of the Bcl-2 family. Quercetin treated cells were analyzed for protein expression of pro-apoptotic Bax and caspase-3, and anti-apoptotic Bcl-2 and Bcl-x_L _proteins by Western blotting with specific antibodies. Protein levels were quantitated by densitometry and expressed as a percentage of the control. As shown in Fig. 2, a strong 21-kDa band representing Bax protein was seen in quercetin treated lysate, compared with a control. We then examined the Bcl-2 and Bcl-x_L _proteins expression in quercetin treated prostate cancer cells, we observed significant decrease of Bcl-2 and Bcl-x_L _proteins (Fig. 2). Western blot analysis was also performed to analyse caspase-3 (active fragment). As shown in figure 2, quercetin increased the levels of caspase-3, suggesting the possible involvement of caspase-3 activation as one of the possible mechanism of apoptosis induction.

### Apoptosis

**Figure 3:**
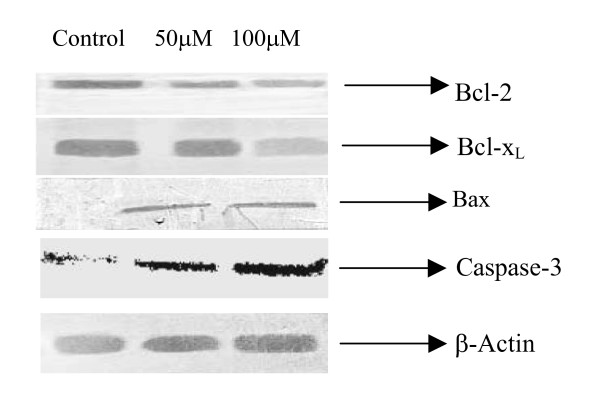
Effects of quercetin on cell cycle distribution in PC-3 cells. PC-3 cells were treated with quercetin at doses of 50 and 100 μM for 24 h in a 25 cm^2 ^flasks. After treatment 1 × 10^6 ^cells were trypsinized and suspended in 1 ml of fluorochrome solution (50 mg/ml propidium iodide, 1 mg/ml RNase A, 1.5% Triton X-100) for at least 1 h in the dark at 4°C. Cell cycle analysis was performed using a Beckman Vantage flow cytometer, and quantitation of cell cycle distribution was performed using Multicycle software (Phoenix Flow Systems, San Diego, CA). Significant decrease in the proportions of cells in G_1_-S and increase in G_2_-M-phase, were evident after exposure to quercetin for 24 h. A concomitant increase of cells in SubG1-phase was observed in quercetin treated cells, indicating induction of apoptosis. The experiments were repeated thrice, *-denotes the statistical significance at *P < 0.05 *level between control and quercetin treatment groups using Student's Newman Keuls (SNK) test.

**Figure 4:**
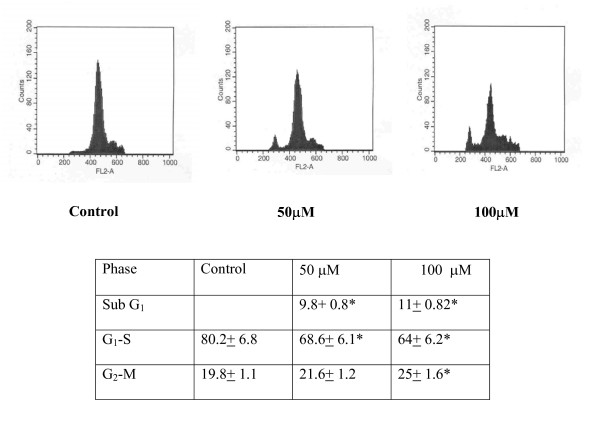
A photograph of an UV light-illuminated agarose gel containing total cellular DNA; lanes were loaded with DNA preparations made from 24 h and 48 h quercetin treated PC-3 cells with different concentrations.

**Figure 5:**
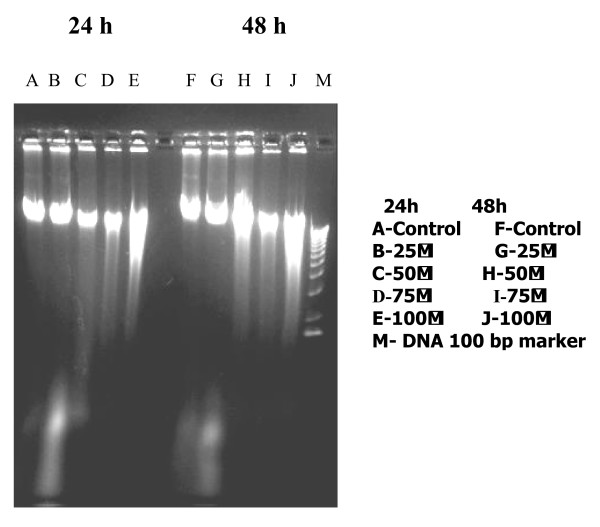
Effect of quercetin on induction of apoptosis in PC-3 cells. The percentage of apoptotic cells was determined and summarized. Generation of free 3'-OH DNA fragments was determined by TUNEL assay. Each value is mean ± SEM. Experiments were carried out in triplicates. *-represents statistical significance between control Vs quercetin treatment groups at *P < 0.05 *level using Student Newman-Keuls (SNK) test.

To analyze the mechanism responsible for the growth inhibition of PC-3 cells by quercetin, the effects of quercetin on programmed cell death was examined. A concomitant increase of cells in Sub G1-phase was observed in quercetin treated cells, indicating induction of apoptosis (Fig. 3). The sub G1 phase showed significant increase in the cell number in quercetin treated when compared with control. Significant decrease in the proportions of cells in G and S phases was evident with increase in G_2 _M phase. DNA agarose electrophoresis for quercetin treated cells showed quercetin treatment caused DNA fragmentation in the PC-3 cells (Fig. 4). Quantitative apoptotic cell death was performed to confirm the induction of apoptosis. Quercetin-caused apoptotic death of PC-3 cells was analysed by TUNEL assay, which showed that 50 and 100 μM treatment of quercetin for 24 h significantly (*P < 0.05*) increased the percentage of apoptotic cells up to 10 fold compared with that of control (Fig. 5).

## Discussion

The insulin like growth factors (IGFs) plays an increasingly recognized role in the autocrine/paracrine regulation of cancer cells. There is now accumulating evidence that IGFBP-3 has an important anti-proliferative and pro-apoptotic role in the regulation of cancer cell growth [27]. However, although it has been described, as an effector of anticancer cancer agents-induced apoptosis, moreover there are no reports available in the role IGFBP-3 in quercetin-induced apoptosisin prostate cancer cells. This study examined the effects of quercetin on the induction of apoptosis and possible mediating role of IGFBP-3. The increased endogenous production ofIGFBP-3 by quercetin was associated with the induction of apoptosis in this human prostate cancer cell line. This induction was observed under serum-free conditions, suggesting possible pro-apoptotic role of IGFBP-3 in this cells. There are reports suggesting that IGFBP-3 as effector of apoptosis inducer in PC-3 cells [7,8,12]. This effect was observed in cells not expressing functional p53 protein, so it was independent of p53. This supports the work by Rajah *et al*. [13] who demonstrated the proapoptotic function of IGFBP-3 in p53 negative prostate carcinoma cells (PC-3) as well as IGFRI deficient mouse fibroblasts.

The induction of apoptosis by quercetin was associated with changes in both pro-apoptotic and anti-apoptotic members of the Bcl-2 family as well as caspase-3. The ratio of pro-apoptotic Bax protein to anti-apoptotic proteins, Bcl-2 and Bcl-x_L _is a crucial determinant of both cellular susceptibility to apoptosis [21]. Normal prostate epithelium expresses both Bax and Bcl-2 [22,23], the levels of which may be regulated by androgen and estrogen and other factors. IGFBP-3 expression might be one of the modulator of the ratio of apoptotic proteins by up-regulating the pro-apoptotic protein, Bax and down-regulating Bcl-2 and Bcl-x_L _proteins in quercetin treated PC-3 cells. There is evidence that anti-apoptotic signaling through the IGFRI is associated with changes in expression of Bcl-2 and Bcl-x_L _[28,29].

By autocrine/paracrine actions IGFs can increase the cell turnover and the susceptibility of malignant cells. It was already reported that IGFs inhibits the programmed cell death [30]. Experiments using animal and cell culture have shown that the antiapoptotic activity of IGF-I is counterbalanced by the activity of IGFBP-3, which may have a direct and independent-stimulating action on apoptosis [31]. The predictive value of IGF-I may be useful in screening for cancer. For example, the ratio of IGF-I to prostate specific antigen may be a better predictor of the development of prostate cancer than the antigen alone [32].

Recent evidences suggest that silibinin, a commonly occurring flavonoid can inhibit the IGF-I secretions, and increase the level of IGFBP-3 and induce of apoptosis in both *in vivo *and *in vitro *model of prostate cancer [33,34]. IGF-I interacts with IGF-IR to stimulate cell growth. Insulin receptor substrate-I (IRS-I) is a key signaling molecule activated by IGFs. Increased IRS-I tyrosine phosphorylation by IGFs is directly correlated with increased activation of the downstream effector molecules phosphatidylinositol 3' kinase and mitogen activated protein kinase, and the growth response to IGFs is mediated by these pathways [35,36]. It was reported that IGFBP-3 reduces the IRS-I tyrosine phorphorylation [34]. The present results demonstrate that quercetin induces IGFBP-3 secretions and that IGFBP-3 reduces the amount of available ligands (IGFs) available for the interaction of IGF-IR. Thus it appears that the antiproliferative action of quercetin in androgen- independent PC-3 cells involves increased IGFBP-3 production and induction of apoptosis. Furthermore, quercetin also deserves study in the context of prostate cancer preventions, because others have recently shown in populations studies that higher circulating IGFs level and/or lowers IGFBP-3 levels are associated with increased risk of prostate cancer [37,38]. It is relevant that the quercetin shown to be nontoxic in humans includes the possibility that this compound may be useful in prostate cancer treatment. There is also evidence that IGFBP-3 can induce apoptosis by itself, as well as potentiate the apoptotic effects of other factors such as ionizing and UV irradiation and chemotherapeutic agents [39-41].

Hence, the present study demonstrates that quercetin is capable of mediating growth inhibition against prostate cancer cells *in vitro *and is pro-apoptotic. As quercetin is able to induce apoptosis in PC-3 cells, the present findings suggest that quercetin-induced apoptosis could be mediated by the down regulation of IGFs and up regulation of IGFBP-3 in PC-3 cells. Therefore, quercetin can be considered for the evaluation of their *in vivo *or clinical efficacy against prostate cancer.

## Conclusion

This data raise the possibility that IGFBP-3 is the positive regulator of quercetin-induced apoptosis in PC-3 cells as IGFBP-3 is reported to induce apoptosis in various cancer cell lines. The increased level of IGFBP-3 was associated with increased pro-apoptotic proteins and apoptosis in response to quercetin, suggesting it may be mediated via its modulation of the Bax: Bcl-2 protein ratio and hypothesize that IGFBP-3 act as an effector of quercetin induced-apoptosis in prostate cancer cells. The role of IGFBP-3 as an effector of p53-independent apoptotic pathways has particular relevance in the treatment of prostate cancer, where inactivating mutations in the p53 gene occur at high frequency. Hence our data raise the possibility that IGFBP-3 is the positive regulator of quercetin-induced apoptosis and quercetin may have some therapeutic value for prostate cancer.

## Note

**Table 1 **Effect of quercetin on IGF-I, -II and IGFBP-3 secretion by the PC-3 cells. Cells were seeded at a density of 1 × 10^6^/cm^2 ^in 100 mm petri dishes and the medium then replaced with quercetin containing medium. After 24 h and 48 h, media were collected and quantified for IGF-I, -II and IGFBP-3. The results shown are those for five separate experiments. *-denotes the statistical significance at *P < 0.05 *level between control and quercetin treatment groups using Student's Newman Keuls (SNK) test. ND – Not detectable.
